# Construction of a Colorimetric and Near-Infrared Ratiometric Fluorescent Sensor and Portable Sensing System for On-Site Quantitative Measurement of Sulfite in Food

**DOI:** 10.3390/foods13111758

**Published:** 2024-06-04

**Authors:** Xiaodong Chen, Chenglu Zhao, Qiwei Zhao, Yunfei Yang, Sanxiu Yang, Rumeng Zhang, Yuqing Wang, Kun Wang, Jing Qian, Lingliang Long

**Affiliations:** 1 Key Laboratory of Modern Agricultural Equipment and Technology (Ministry of Education), Jiangsu University, Zhenjiang 212013, China; 2 School of Chemistry and Chemical Engineering, Jiangsu University, Zhenjiang 212013, China

**Keywords:** sulfite, fluorescent sensor, on-site measurement, food sample, portable sensing system

## Abstract

Sulfites play imperative roles in food crops and food products, serving as sulfur nutrients for food crops and as food additives in various foods. It is necessary to develop an effective method for the on-site quantification of sulfites in food samples. Here, 7-(diethylamino) quinoline is used as a fluorescent group and electron donor, alongside the pyridinium salt group as an electron acceptor and the C=C bond as the sulfite-specific recognition group. We present a novel fluorescent sensor based on a mechanism that modulates the efficiency of intramolecular charge transfer (ICT), **CY**, for on-site quantitative measurement of sulfite in food. The fluorescent sensor itself exhibited fluorescence in the near-infrared light (NIR) region, effectively minimizing the interference of background fluorescence in food samples. Upon exposure to sulfite, the sensor **CY** displayed a ratiometric fluorescence response (I_447_/I_692_) with a high sensitivity (LOD = 0.061 μM), enabling accurate quantitative measurements in complex food environments. Moreover, sensor **CY** also displayed a colorimetric response to sulfite, making sensor **CY** measure sulfite in both fluorescence and colorimetric dual-signal modes. Sensor **CY** has been utilized for quantitatively measuring sulfite in red wine and sugar with recoveries between 99.65% and 101.90%, and the RSD was below 4.0%. The sulfite concentrations in live cells and zebrafish were also monitored via fluorescence imaging. Moreover, the sulfite assimilated by lettuce leaves was monitored, and the results demonstrated that excessive sulfite in leaf tissue could lead to leaf tissue damage. In addition, the sulfate-transformed sulfite in lettuce stem tissue was tracked, providing valuable insights for evaluating sulfur nutrients in food crops. More importantly, to accomplish the on-site quantitative measurement of sulfite in food samples, a portable sensing system was prepared. Sensor **CY** and the portable sensing system were successfully used for the on-site quantitative measurement of sulfite in food.

## 1. Introduction

Sulfur is known as the fourth most essential macronutrient required for food crop growth after nitrogen, potassium, and phosphorus. A deficiency of sulfur nutrients in food crops can lead to severe adverse effects on crop yield and quality [1,2,3,4]. As derivatives of sulfur dioxide (SO_2_), sulfites (HSO_3_^−^ or SO_3_^2−^) play an imperative role in the balance of sulfur nutrients in food crops. After being taken up by the root or leaf, the sulfites in food crops are reduced to sulfide by sulfite reductase (SIR), followed by synthesizing cysteine under the catalysis of cysteine synthetase, and are further converted into organic sulfur compounds, which are finally used as sulfur nutrients [5,6]. The sulfite concentration can serve as an important indicator for evaluating sulfur nutrition levels in food crops. On the other hand, sulfites are extensively used as additives in food products to prevent microbial spoiling, oxidation, and browning reactions during food production and storage [7,8,9]. However, excessive sulfites in food can cause irreversible damage to cells and tissues in consumers’ bodies, which leads to many diseases such as respiratory diseases, allergic reactions, cardiovascular diseases, asthmatic reactions, and even lung cancer [10,11]. Due to their potential detrimental effects, the United States Food and Drug Administration (FDA) requires a product to be labeled if it contains 10 ppm of sulfites [12]. The World Health Organization (WHO) recommends a daily intake of sulfites ranging from 0 to 0.7 mg/kg (equivalent to 0–49 mg for a 70 kg adult) [13]. In view of the significant roles of sulfites in food crops and food products, it necessary to develop an effective method for the on-site quantification of sulfites in food samples.

At present, many analytical methods have been established to detect sulfite, such as the electrochemical method [14], flow injection [15], capillary electrophoresis [16], spectrophotometry [17], and high-performance liquid chromatography (HPLC) [18]. Nevertheless, these traditional analytical methods are usually completed by professional technicians in well-equipped laboratories, making them unsuitable for on-site quantitative measurement. In recent years, colorimetric and fluorescence analytical methods have attracted more and more attention because their signal output can be readily obtained by unaided eyes with no need for expensive equipment and cumbersome operations, making them beneficial for on-site measurement [19,20,21,22]. Up to now, some elegant fluorescent sensors for sulfite have been explored based on the specific nucleophilic reactions of sulfite with electron-deficient groups, including aldehydes [23,24], C=N [25,26], levulinic acid [27,28], azo groups [29,30], C=C [31,32], etc. However, the fluorescent sensors that are capable of quantitatively measuring sulfite in food samples on-site remains scarce. To on-site quantitatively measure sulfite in food, fluorescent sensors need to have the following characteristics: (1) The fluorescent sensors preferably respond to sulfite in the near-infrared (NIR) fluorescence range (650–900 nm), as the fluorescence in this range might reduce the interference of autofluorescence in food samples [33,34,35]. (2) The fluorescent sensors should exhibit a ratiometric fluorescence response to sulfite as it could effectively eliminate the fluorescence intensity errors originating from sensor distribution, excitation light intensity, photobleaching [36,37,38], etc. (3) Fluorescent sensors are better for displaying colorimetric responses to sulfite, because both fluorescence and colorimetric dual-signal responses to sulfite will make the measurement more reliable [39,40,41,42]. Therefore, the development of a colorimetric and NIR fluorescent sensor for the on-site quantitative measurement of sulfite in food samples is in great demand.

**Figure 1:**
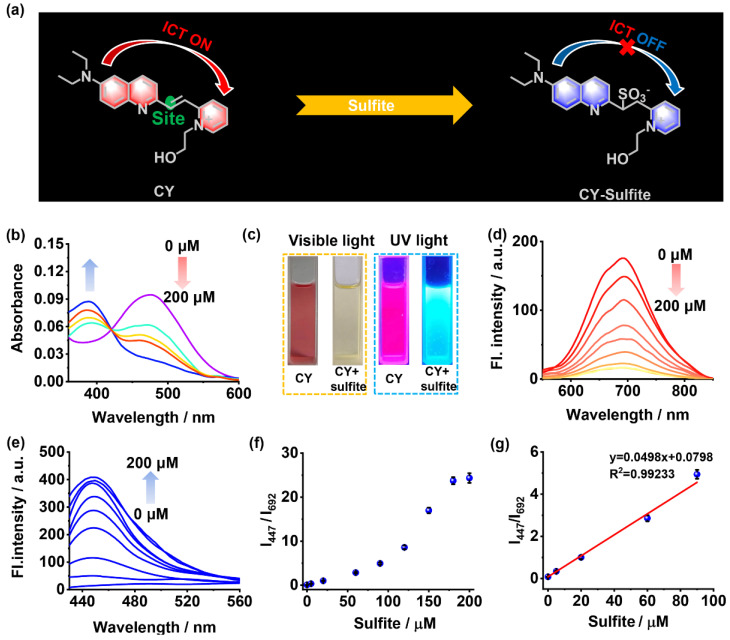
(**a**) Response mechanism of sensor **CY** to sulfite. (**b**) The absorption spectra of sensor **CY** (5 μM) with sulfite (0–200 μM). (**c**) The photo of sensor **CY** with or without sulfite under visible light and UV lamp. (**d**) The fluorescence spectra of sensor **CY** (5 μM) with sulfite (0–200 μM), λ_ex_ = 500 nm. (**e**) The fluorescence spectra of sensor **CY** (5 μM) with sulfite (0–200 μM), λ_ex_ = 400 nm. (**f**) The relationship of fluorescence intensity ratios (I_447_/I_692_) and sulfite concentrations (0–200 μM). (**g**) The linearity of fluorescence ratios (I_447_/I_692_) and sulfite concentrations (0–90 μM).

Herein, a colorimetric and NIR fluorescent sensor, **CY**, for selectively measuring sulfite was rationally developed, and its applicability in food was explored. In sensor **CY**, the electron-donating 7-diethylamino-azacoumarin fluorophore was conjugated with an electron-withdrawing pyridium moiety via a C=C bond to form a large conjugated structure (Figure 1a). An efficient intramolecular charge transfer (ICT) process should be occurred between the two moieties, which makes the fluorescence of sensor **CY** fall into the NIR range. When sensor **CY** was treated with sulfite, the sulfite underwent a selective nucleophilic addition reaction with the C=C bond in sensor **CY**. As a result, the conjugation between the 7-diethylamino-azacoumarin fluorophore and the pyridium moiety was broken, and the ICT process was inhibited. Therefore, sensor **CY** could exhibit an NIR ratiometric fluorescence response to sulfite. In addition, the significant inhibition of the ICT process renders sensor **CY** able to display a colorimetric response to sulfite. Accordingly, sensor **CY** was able to respond to sulfite in dual-signal mode. Sensor **CY** has been applied for quantitatively measuring sulfite in red wine and crystal sugar. The sulfite concentrations in live cells and zebrafish were also tracked by fluorescence imaging. Moreover, the variations in sulfite levels in lettuce leaf tissue and lettuce stem tissue were monitored. To accomplish the on-site quantitative measurement of sulfite in food samples, a portable sensing system was prepared. Sensor **CY** and the portable sensing system were successfully employed for the on-site quantitative measurement of sulfite in food.

## 2. Materials and Methods

### 2.1. Materials and Reagents

All the reagents used in the experiments were directly purchased from commercial suppliers. The organic solvents were purified and dried by standard methods prior to use. The water used throughout the experiments was distilled twice. TLC analyses were conducted on silica gel plates and column chromatography was carried out over silica gel (mesh 200–300). The silica gel plates and silica gel were purchased from Qingdao Ocean Chemicals. The white 380 nm and 480 nm LEDs were purchased from Youjing optoelectronics. The optical filters were purchased from Fuzhe.

### 2.2. Sensor Synthesis and Characterization

Compound 2 (100 mg, 0.44 mmol) and compound 3 (150 mg, 0.678 mmol) were mixed in ethanol (5 mL). Then, piperidine (10 μL) was added to the mixture. The reaction mixture was heated to reflux and stirred for 0.5 h. After cooling to room temperature, the solvent was evaporated in vacuo. The residue was purified by column chromatography (dichloromethane/methanol = 100:1, *v*/*v*) to obtain compound **CY** as a red solid (35.3 mg, yield: 18.8%); 1H NMR (CDCl_3_, 400 MHz), δ (ppm): 9.52 (s, 1H), 8.32 (d, J = 8.0 Hz, 1H), 8.25 (d, J = 8.4 Hz, 1H), 7.97 (m, 3H), 7.84 (m, 1H), 7.76 (m, 2H), 7.38 (dd, J = 9.6 Hz, 2.8 Hz, 1H), 6.76 (d, J = 2.8 Hz, 1H), 5.21 (s, 1H), 4.24 (s, 1H), 3.55 (q, J = 6.8 Hz, 4H), and 1.31 (t, J = 6.8 Hz, 6H); HRMS (ESI) *m*/*z* calculated for C_22_H_26_N_3_O [M]+: 348.2070; found: 348.2065.

### 2.3. Quantitative Measurements of Sulfite in Red Wine and Sugar by Fluorescence Spectra

Red wine and sugar were purchased from local supermarkets and used as purchased. The red wine was diluted 50 times with PBS solution (pH 7.4) and used as the red wine analyte solution. The sugar was dissolved in PBS solution (pH = 7.4), and its concentration was maintained at 0.05 g/mL, which was used as the sugar analyte solution. Subsequently, 0.05 mL of the **CY** stock solution, 1.95 mL of DMSO, and 3 mL of the analyte solution (red wine analyte solution or sugar analyte solution) were added into a 5.0 mL volumetric flask. The mixed solution was diluted to the mark with 10 mM potassium phosphate buffer (pH = 7.4). After being incubated for 5 min, the fluorescence spectra were measured, and the fluorescence ratios (I_447_/I_692_) were obtained. According to the equation in Figure 1g, the concentrations of sulfite were determined. The red wine analyte solution or sugar analyte solution were further spiked with different concentrations of sulfite (10 and 20 μM), and then the concentrations of sulfite in these analyte solutions were determined according to the above method.

### 2.4. On-Site Quantitative Measurement of Sulfite in Tomato Leaves by Sensor **CY** in Combination with Self-Made Portable Sensing System

The tomato plant leaves (2 g) were chopped, grinded, and then extracted with PBS buffer (40 mL). The resulting solution was centrifugated at 5000 r/min for 20 min. The supernatant (3 mL) and sensor **CY** (5 × 10^−4^ M) stock solution (0.2 mL) were transferred into a 5.0 mL volumetric flask. The mixed solution was diluted to the mark with DMSO. The obtained solution was sufficiently shaken and kept at room temperature for 5 min. After that, the solution (2 mL) was transferred into a Petri dish. The colorimetric detection and fluorescence detection by the portable sensing system were conducted as the above similar procedure in aqueous solution. According to the calibration curve, the sulfite content in tomato leaves was determined by the colorimetric method. Moreover, according to the calibration curve, the sulfite content in tomato leaves was determined by the fluorescence method.

In addition, the extract of tomato plant leaves was further spiked with different concentrations of sulfite (30, 60 and 90 μM), and the sulfite contents were determined by the colorimetric method and fluorescence method.

## 3. Results

### 3.1. Optical Responses of Sensor **CY** to Sulfite

The optical properties of sensor **CY** to sulfite were investigated in PBS solution (10 mM, pH 7.4, with 40% DMSO as co-solvent). As shown in Figure 1b, sensor **CY** exhibited an intensive and broad absorption band centered at 477 nm, which is apparently ascribed to the ICT transition between the 7-diethylamino-azacoumarin fluorophore and the pyridium moiety. When increasing amounts of sulfite were added, the absorption peak at 477 nm was gradually attenuated, while a new absorption peak at 386 nm emerged and became enhanced, indicating that the ICT process in sensor **CY** was inhibited. Moreover, the changes in absorption spectra made the solution color under visible light display an obvious change from red to light yellow (Figure 1c). Thus, sensor **CY** showed a colorimetric response to sulfite. Sensor **CY** itself exhibited intense fluorescence at 692 nm, which is in the NIR light range (Figure 1d). Upon treating with sulfite, the fluorescence at 692 nm decreased (Figure 1d), while a new fluorescence at 447 nm appeared and increased (Figure 1e). Concurrently, the visual fluorescence color changed from rose red to light blue under UV light (Figure 1c). The changes in fluorescence spectra made the fluorescence intensity ratio (I_447_/I_692_) enhance drastically with the concentration of sulfite (Figure 1f). When 200 μM of sulfite was added, the fluorescence ratio (I_447_/I_692_) reached a plateau (Figure 1f). Moreover, a good linear relationship (R^2^ = 0.9923) between fluorescence intensity ratios (I_447_/I_692_) and sulfite concentrations within the range of 0.203–90 μM was observed (Figure 1g), manifesting that sensor **CY** was capable of quantitatively detecting sulfite. Sensor **CY** exhibited a highly sensitive response to sulfite, and the detection limit of sensor **CY** toward sulfite was as low as 0.061 μM (S/N = 3).

**Figure 2:**
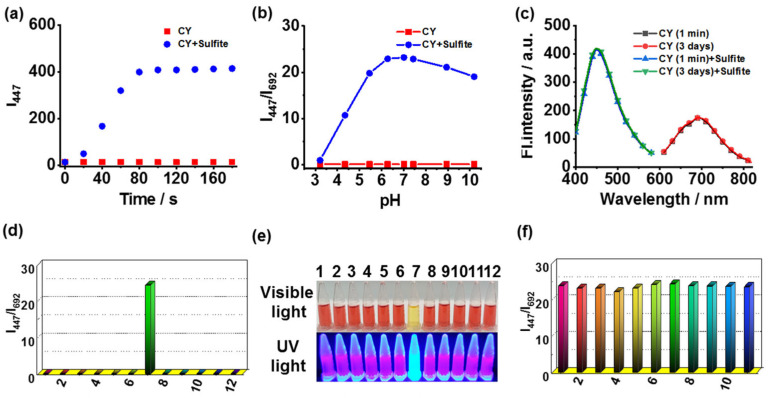
(**a**) The time-dependent fluorescence intensity (I_447_) of sensor **CY** without and with sulfite. (**b**) The fluorescence ratios (I_447_/I_692_) of sensor **CY** (5 μM) under various pH conditions with or without sulfite (200 μM). (**c**) The fluorescence spectra of the sensor **CY** (5 μM) in testing solution for 1 min and 3 days. (**d**) The fluorescence ratio (I_447_/I_692_) of sensor **CY** (5 μM) in the presence of various analytes (200 μM): (1) blank; (2) Cl^−^; (3) Mg^2+^; (4) Fe^3+^; (5) Ala; (6) Glu; (7) SO_3_^2−^; (8) Cys; (9) GSH; (10) H_2_S; (11) HClO; and (12) H_2_O_2_. (**e**) Image of sensor **CY** (5 μM) with different analytes (200 μM) under visible light and UV light. (**f**) The anti-disturbance ability of sensor **CY** (5 μM) toward sulfite (200 μM) in the presence of different analytes (200 μM): (1) blank; (2) Cl^−^; (3) Mg^2+^; (4) Fe^3+^; (5) Ala; (6) Glu; (7) Cys; (8) GSH; (9) H_2_S; (10) HClO; and (11) H_2_O_2_.

The time-dependent fluorescence responses of **CY** toward sulfite were studied. The reaction of **CY** to sulfite could be completed within 90 s (Figure 2a). Therefore, sensor **CY** was able to detect sulfite in real time. In addition, the fluorescence response of **CY** toward sulfite at different pH levels was tested. The fluorescence ratio (I_447_/I_692_) displayed a pronounced enhancement to sulfite in the range of pH 5.45–10.19 (Figure 2b). Thus, sensor **CY** could sense sulfite in physiological and weak alkaline conditions. Sensor **CY** was stable in testing solution for at least 3 days (Figure 2c). The selectivity and anti-disturbance capability of sensor **CY** toward sulfite were also evaluated. Sensor **CY** was treated with a range of representative species, including Cl^-^, Mg^2+^, Fe^3+^, Ala, Glu, SO_3_^2−^, Cys, GSH, H_2_S, HClO, and H_2_O_2_. The results revealed that only sulfite induced significant changes in the fluorescence ratio (I_447_/I_692_) (Figure 2d). Furthermore, the selective response of **CY** to sulfite can be visualized by naked eyes under visible light and UV light (Figure 2e), enabling sensor **CY** to detect sulfite on-site without requiring expensive and complicated equipment. Moreover, the anti-disturbance studies signified that sensor **CY** could effectively respond to sulfite even in the presence of interfering species (Figure 2f). These results demonstrated that sensor **CY** held robust selectivity for sulfite detection.

### 3.2. The Fluorescence Sensing Mechanism of the Sensor toward Sulfite

**Figure 3:**
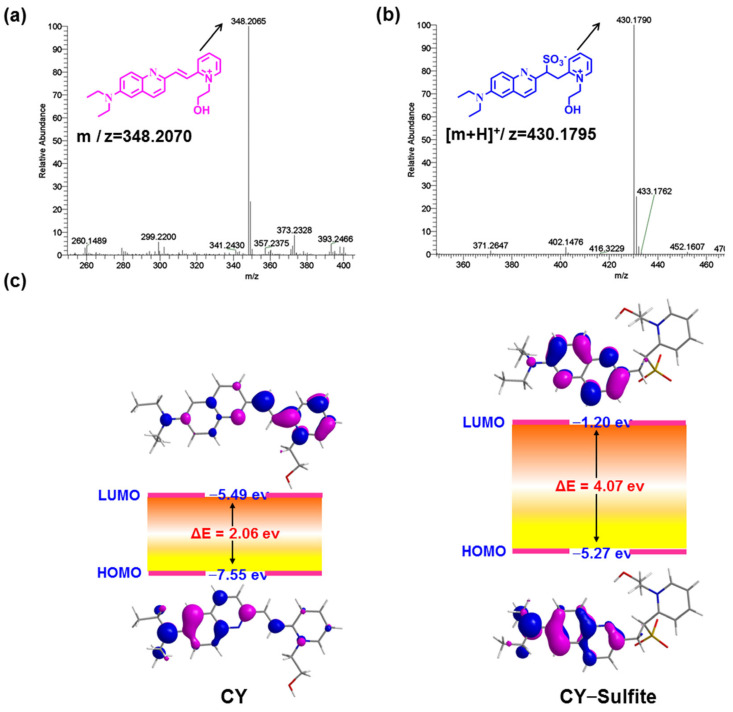
(**a**) The HRMS spectra of sensor **CY.** (**b**) The HRMS spectra of sensor **CY** reacting with sulfite. (**c**) Schematic diagram of LUMO and HOMO orbitals and energy levels of compound **CY** and **CY–Sulfite**.

As sulfite readily undergoes a nucleophilic addition reaction with electron-deficient C=C, sensor **CY** will be converted to **CY–Sulfite** in the presence of sulfite (Figure 1a). To confirm this, sensor **CY** with and without sulfite was analyzed by HRMS. Sensor **CY** itself exhibited a main peak at 348.2065 (Figure 3a). However, after treating **CY** with sulfite, a main peak was found at 430.1790, which is assigned to **CY–Sulfite** (Figure 3b), in agreement with the design. To further inquire the underlying optical response mechanism of sensor **CY** to sulfite, sensor **CY** and its reaction product **CY–Sulfite** were studied by time-dependent density functional theory (TD-DFT). As illustrated in Figure 3c, an efficient ICT process could be found from the 7-diethylamino-azacoumarin fluorophore to the pyridium moiety in sensor **CY**, which was responsible for the absorption and fluorescence spectra of sensor **CY**. However, in **CY–Sulfite**, the electron in HOMO and LUMO was mainly distributed in the 7-diethylamino-azacoumarin moiety. Hence, the ICT process was inhibited, which resulted in the absorption and fluorescence spectra being blue-shifted compared with that of sensor **CY**. Therefore, the optical response of sensor **CY** to sulfite was theoretically revealed.

### 3.3. Detection of Sulfite in Red Wine and Sugar

Sulfite has been extensively employed as an additive in foods. However, immoderate intake of sulfite will bring about health hazards. Thus, sensor **CY** was applied for determining sulfite in commercially available foods such as red wine and crystal sugar. Sensor **CY** was treated with an aqueous solution of red wine and crystal sugar, and the fluorescence ratios (I_447_/I_692_) were obtained. According to the equation in Figure 1g, the concentrations of sulfite were determined. As shown in Table 1, the content of sulfite in red wine and crystal sugar was detected to be 3.39 μM and 4.95 μM, respectively. Moreover, sensor **CY** was capable of determining sulfite spikes with recoveries between 99.65% and 101.90%, and the RSD was below 4.0%. Thus, sensor **CY** could be used to quantitatively determine sulfite in real foods.

### 3.4. Fluorescence Imaging of Sulfite in Live Cells and Zebrafish

**Figure 4:**
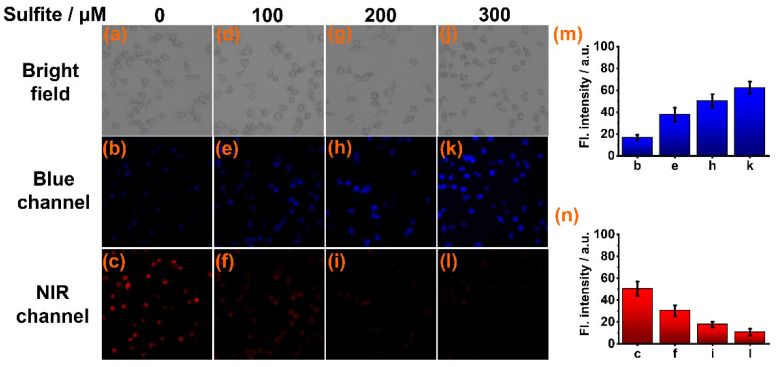
Confocal fluorescence imaging of sensor **CY** (10 μM) in Caki-1 cells incubated with different concentrations of sulfite: (**a**–**c**) 0 μM sulfite; (**d**–**f**) 100 μM sulfite; (**g**–**i**) 200 μM sulfite; (**j**–**l**) 300 μM sulfite. (**m**) Average fluorescence intensities in blue channel. (**n**) Average fluorescence intensities in NIR channel.

To test its ability to detect sulfite in biological milieus, sensor **CY** was used for imaging sulfite in live cells via a laser confocal fluorescence microscope. Firstly, the cytotoxicity of **CY** to live Caki-1 cells was evaluated by an MTT assay. The results indicated that sensor **CY** has very low cellular toxicity (Figure S1). Then, the Caki-1 cells were stained with sensor **CY** (10 μM) for 30 min. Intense NIR fluorescence (Figure 4c) and weak blue fluorescence (Figure 4b) were observed. When the sensor **CY**-stained cells were further treated with increasing amounts of sulfite (100, 200, and 300 μM), the NIR fluorescence decreased (Figure 4n), while the blue fluorescence increased (Figure 4m). These findings confirm that sensor **CY** can effectively detect sulfite in live cells.

**Figure 5:**
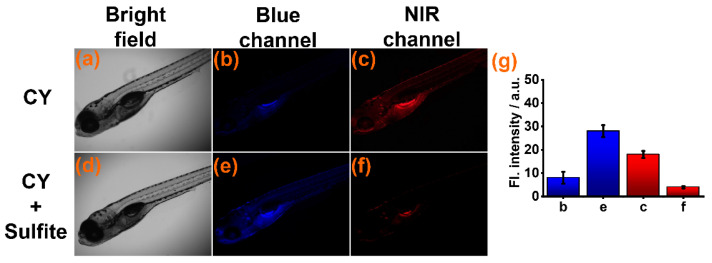
(**a**–**c**) Fluorescence imaging of zebrafish treated with sensor **CY** (10 μM). (**d**–**f**) Fluorescence imaging of zebrafish pre-treated with sensor **CY** (10 μM), then treated with sulfite (200 μM). (**g**) Average fluorescence intensities in blue and NIR channel.

Subsequently, sensor **CY** was used for detecting sulfite in zebrafish. The zebrafish were cultured in aqueous solution with sensor **CY** (10 μM) for 60 min. Weak fluorescence was found in the blue channel (Figure 5b) and strong fluorescence was seen in the NIR channels (Figure 5c). When **CY**-stained zebrafish were further cultured in aqueous solution with sulfite, the fluorescence intensity in the NIR channel appeared to decrease (Figure 5f), but the fluorescence intensity in the blue channel was drastically increased (Figure 5e). These experimental results indicated that sensor **CY** was suitable for sulfite detection in a live animal environment.

### 3.5. Fluorescence Imaging of Sulfite in Food Crops

**Figure 6:**
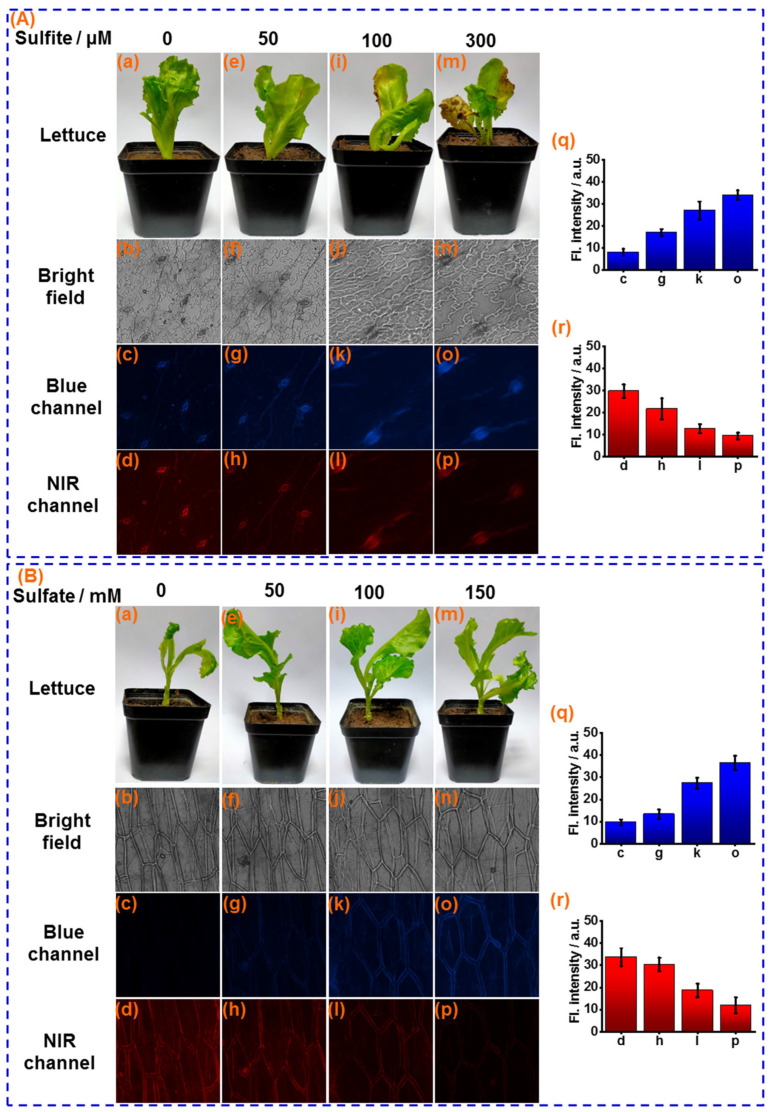
(**A**) (**a**,**e**,**i,m**): The photo of lettuce leaves after sprayed with 0, 50, 100 and 300 μM sulfite, respectively. Confocal fluorescence images of sensor **CY** (10 μM) stained lettuce leaf tissues after spayed with different concentration of sulfite for 5 days: (**b**–**d**) 0 μM; (**f**–**h**) 50 μM; (**j**–**l**) 100 μM and (**n**–**p**) 200 μM, respectively; (**q**) Average fluorescence intensities of blue channel; (**r**) Average fluorescence intensities of NIR channel. (**B**) (**a,e**,**i,m**): The photo of sensor **CY** stained lettuce tissue after cultivated with 0, 50 mM, 100 mM and 150 mM sulfate, respectively. Confocal fluorescence images of sensor **CY** (10 μM) stained lettuce leaf tissues after cultivated with different concentration of sulfate for 7 days: (**b**–**d**) 0 mM; (**f**–**h**) 50 mM; (**j**–**l**) 100 mM and (**n**–**p**) 150 mM, respectively; (**q**) Average fluorescence intensities of blue channel; (**r**) Average fluorescence intensities of NIR channel.

Atmospheric sulfur dioxide (SO_2_) can be easily hydrated to form sulfites on the leaf surface of food crops and be used as a source of sulfur nutrients. However, as a strong nucleophile, over-loaded sulfite on the leaf surface will deleteriously affect crop health, with effects such as leaf chlorosis, necrosis, and long-term yield reduction. Therefore, sensor **CY** was further applied for measuring sulfite in the leaf of lettuce, a representative vegetable crop. The lettuce leaves were sprayed with different concentrations of sulfite solution (0, 50, 100, 300 μM) every day. After 5 days, significant chlorosis was observed when the lettuce leaves were sprayed with 100 and 300 μM of the sulfite solution (Figure 6A(i,m)), which is one of unique forms of sulfite damage to crops. The lettuce leaf tissues were stained with sensor **CY** and were subjected to fluorescence imaging. As displayed in Figure 6A(j,n), the sulfite-induced lettuce leaf damage can be clearly observed by bright-field images. Moreover, as the concentration of sulfite solution increased during spraying, more intensive fluorescence in the blue channel and weaker fluorescence in the NIR channel were observed (Figure 6A(q,r)). These results demonstrate that the concentration of sulfite assimilated into leaf tissues is dependent on the sulfite concentration on the surface of the leaf, and excessive sulfite in leaf tissue could lead to leaf tissue damage.

Sulfur is the fourth most essential macronutrient for food crops, which is highly associated with crop growth and quality. Crop roots actively take up sulfate and transport it to the stems and leaves via the xylem. Then, the sulfate is activated to become 5′-phosphoadenosine sulfate (APS), which is reduced to sulfite and subsequently assimilated to sulfur nutrients. Therefore, the sulfite functions as an important intermediate for sulfur nutrients. The sulfite concentration in food crops can be considered to be an indicator for sulfur nutrients in food crops. Hence, sensor **CY** was utilized for measuring changes in sulfite levels in lettuce stems cultivated with different amounts of sulfate. As shown in Figure 6B(q,r), when the lettuces were cultivated with incremental amounts of sulfate (0, 50, 100, and 150 mM) for 7 days, the growth trend of lettuce was increased (Figure 6B(a,e,i,m)), as sulfate provides a source of sulfur for lettuce growth. Then, the lettuce stems were sliced and stained with the sensor **CY** for fluorescence imaging. As shown in Figure 6B(q,r), with the increased dosage of sulfate during lettuce growth, the fluorescence in the NIR channel was decreased. Simultaneously, the fluorescence intensity in the blue channel was increased, suggesting that sulfate can be taken up by lettuce roots and further converted to sulfite. Thus, the transformation process of sulfur nutrients has been successfully monitored. These results demonstrated that the sulfite levels might be employed for evaluating sulfur nutrients in food crops.

### 3.6. On-Site Detection of Sulfite in Real Foods

**Figure 7:**
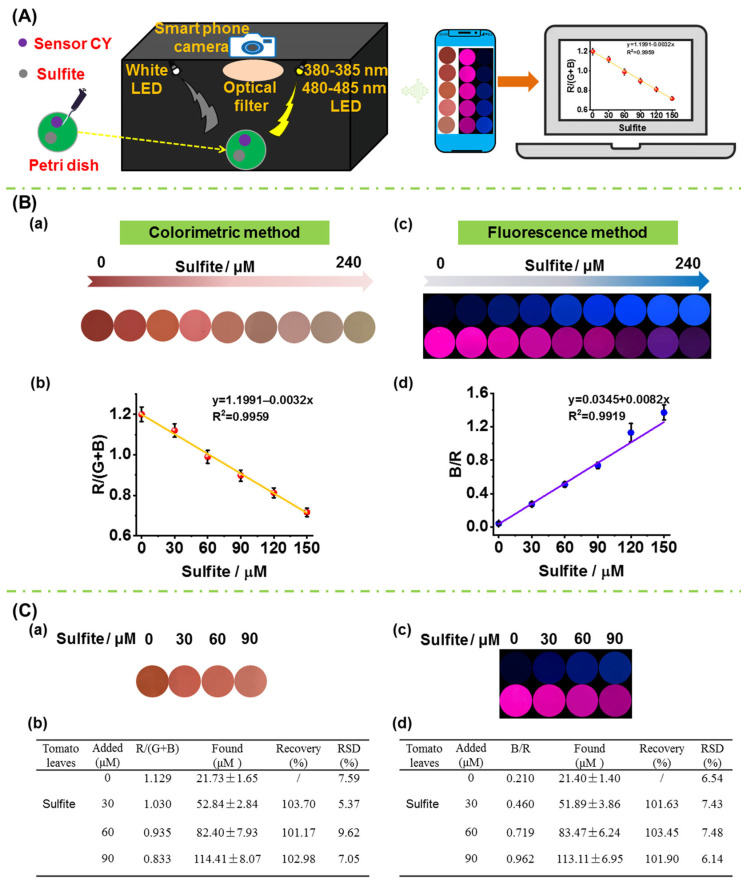
(**A**) A schematic picture of the portable sensing system. (**B**) (**a**) Colorimetric response of sensor **CY** to sulfite (0–240 μM) in PBS buffer solution captured by portable sensing system; (**b**) the linearity of the R/(G + B) value with sulfite concentrations in panel (**a**); (**c**) fluorescence response of sensor **CY** to sulfite (0–240 μM) in PBS buffer captured by portable sensing system; (**d**) the linearity of the B/R value with sulfite concentration in panel (**c**). (**C**) Measurement of sulfite content in tomato leaf by sensor **CY** and portable sensing system via colorimetric method and fluorescence method, respectively; (**a**): the changes in signal outputs of sensor **CY** with tomato leaf extract spiked with increasing contents of sulfite; (**b**) the determined sulfite content in tomato leaf extract; (**c**) the changes in signal outputs of sensor **CY** with tomato leaf extract spiked with increasing content of sulfite; (**d**) the determined sulfite content in tomato leaf extract.

To accomplish on-site quantitative measurement of sulfite in foods, a portable sensing system was fabricated (Figure 7A), and the fabrication details are shown in the supporting information. Firstly, the performance of the sensing system together with sensor **CY** in detecting sulfite in aqueous solution was evaluated. Sensor **CY** in aqueous solution was treated with different concentration of sulfite. Then, the solution was transferred to a Petri dish. For the colorimetric detection, a white LED light was used as the excitation source. And the colorimetric images were directly captured by a smartphone camera. As seen in Figure 7B(a), upon treatment with increasing amounts of sulfite, the solution color changed gradually from red to light yellow. The red intensity values analyzed by Photoshop CS6 software gradually decreased when increasing amounts of sulfite were introduced. Moreover, a good linear correlation (R^2^ = 0.9959) between R/(G + B) and sulfite concentrations (0–150 μM) was observed (Figure 7B(b)), which could be utilized for the quantitative detection of the sulfite contents in crops. The 380–385 nm LED light was employed as the excitation source for blue fluorescence detection. The blue fluorescence images were captured by a smartphone camera through a 420–480 nm optical filter. The 480–485 nm LED light was employed as the excitation source for red fluorescence detection. The red fluorescence images were captured by a smartphone camera through a 680–700 nm optical filter. As shown in Figure 7B(c), as the concentration of sulfite increased, the blue fluorescence was enhanced, while the red fluorescence was attenuated. After being analyzed by Photoshop software, the blue and red fluorescence intensity ratios (B/R) were obtained. The B/R values were enhanced when increasing concentrations of sulfite were introduced (Figure 7B(d)). Moreover, a good linearity (R^2^ = 0.9919) was found between B/R values and sulfite concentrations (0–150 μM). The above results demonstrated that the portable sensing system and sensor **CY** could perform colorimetric and fluorescence dual-mode detection of sulfite.

In order to verify the practicability in real crop samples, the sensor **CY** as well as the portable sensing system were used for the on-site quantitative measurement of sulfite in tomato leaf. The tomato plants were cultivated with sulfate for a week. The tomato plant leaves were chopped, grinded, and then extracted with PBS buffer. The resulting solution was centrifuged. After the supernatant was incubated with sensor **CY** in a Petri dish, the colorimetric images and fluorescence images were captured by the portable sensing system (Figure 7C(a,c)). The colorimetric images were analyzed by Photoshop software, and the R/(G + B) values were obtained. According to the calibration curve in Figure 7B(b), the sulfite content in tomato leaves was determined to be 21.73 μM by the colorimetric method. Moreover, the fluorescence images were also analyzed by Photoshop software, and the B/R values were obtained. According to the calibration curve in Figure 7B(d), the sulfite content in tomato leaves was determined to be 21.40 μM by the fluorescence method, which is similar to that obtained by the colorimetric method. In addition, the extract of tomato plant leaves was further spiked with different concentrations of sulfite (30, 60 and 90 μM), and the sulfite contents were determined by the colorimetric method and fluorescence method. As shown in Figure 7C(b,d), the average recoveries were between 101.17% and 103.70%, and the relative standard derivative (RSD) was determined to be below 10.0%. These results establish that sensor **CY** in combination with the portable sensing system can be employed to quantitatively measure sulfite in crops on-site.

## 4. Conclusions

In summary, we have developed a novel colorimetric and NIR ratiometric fluorescent sensor, **CY**, for the on-site quantitative measurement of sulfite in food. When sensor **CY** was treated with sulfite, the sulfite underwent a nucleophilic addition reaction with the C=C bond in **CY**, resulting in the inhibition of its ICT process. As a result, sensor **CY** exhibited an NIR ratiometric fluorescent response. Moreover, sensor **CY** also displayed a distinct color change from red to light yellow under visible light and from rose red to blue under UV light, indicating that sensor **CY** could measure sulfite by the naked eye in dual-signal mode. Sensor **CY** showed high selectivity and excellent sensitivity to sulfite. The detection limit was measured to be 0.061 μM. Sensor **CY** has been utilized to quantitatively determine sulfite in red wine and sugar with good recoveries. Also, the changes in sulfite concentration in live cells and zebrafish were monitored via fluorescence imaging. Moreover, the sulfite levels in lettuce leaf tissue and stem tissue were tracked. To accomplish the on-site quantitative measurement of sulfite in food samples, a portable sensing system was prepared. Sensor **CY** and the portable sensing system were successfully used for the on-site quantitative measurement of sulfite in food. We expect that the method constructed in this work could be widely used for the on-site quantitative measurement of sulfite in various foods. This research work may also contribute to the development of analytical methods for the on-site quantitative detection of other important substances in foods.

## Figures and Tables

**Table 1:** The sulfite concentration in red wine and crystal sugar determined by sensor **CY** (*n* = 3).

Sample	Added (μM)	Found (μM)	Recovery (%)	RSD (%)
Red wine	0	3.39 ± 0.10	/	2.95
	10	13.58 ± 0.39	101.90	2.87
	20	23.32 ± 0.74	99.65	3.17
Crystal sugar	0	4.95 ± 0.09	/	1.82
	10	14.93 ± 0.44	99.80	2.95
	20	25.13 ± 0.77	100.90	3.06

## Data Availability

The original contributions presented in the study are included in the article and supplementary materials, further inquiries can be directed to the corresponding author.

## Supplementary Materials

The following supporting information can be downloaded at https://www.mdpi.com/article/10.3390/foods13111758/s1, Scheme S1: The synthetical procedures for compound **CY**, Figure S1: MTT results of Caki-1 cell viabilities after incubation with different concentrations of sensor **CY** for 24 h. Data are expressed as mean ± SD (experiment times n = 3); Figure S2: The ^1^H NMR spectra of **CY** recorded in CDCl_3_; Figure S3: A photo of the self-made portable sensing system; Figure S4: The fluorescence spectra (λ_ex_ = 410 nm) of sensor **CY** (5 μM) treated with increasing amounts of sulfite (0–200 μM); Table S1: Comparison of the fluorescent sensors for sulfite detection in food analysis. Figure S4: The fluorescence spectra (λ_ex_ = 410 nm) of sensor CY (5 μM) treated with increasing amounts of sulfite (0-200 μM), Scheme S1: The synthetical procedures for compound CY.

## References

[1] Sharma R.K., Cox M.S., Oglesby C., Dhillon J.S. (2024). Revisiting the role of sulfur in crop production: A narrative review. J. Agric. Food Res..

[2] Chandra N., Pandey N. (2016). Role of sulfur nutrition in plant and seed metabolism of Glycine maxL. J. Plant Nutr..

[3] Prystupa P., Gutierrez-Boem F., Sadras V. (2022). Crop growth rate during the critical period is associated with grain number under sulfur deficiency in barley crops subjected to different levels of nitrogen availability. Crop Pasture Sci..

[4] Thangasamy A., Gorrepati K., Ghodke P.H., Tp S.A., Jadhav M., Banerjee K., Singh M. (2021). Effects of sulfur fertilization on yield, biochemical quality, and thiosulfinate content of garlic. Sci. Hortic..

[5] Chan K.X., Phua S.Y., Van Breusegem F., Kopriva S. (2019). Secondary sulfur metabolism in cellular signalling and oxidative stress responses. J. Exp. Bot..

[6] Nakai Y., Maruyama-Nakashita A. (2020). Biosynthesis of Sulfur-Containing Small Biomolecules in Plants. Int. J. Mol. Sci.

[7] Zhong Z., Li G., Zhu B., Luo Z., Huang L., Wu X. (2012). A rapid distillation method coupled with ion chromatography for the determination of total sulphur dioxide in foods. Food Chem..

[8] Kim H.-J. (1995). Inhibition of Enzymatic Browning Reaction by Sulfite. J. Chem. Educ..

[9] Wu W., Fu G., Xuan R., Zhai L., Lu Y., Tang M., Liu J., Zhang C., Chen H., Wang F. (2022). Food additive sodium bisulfite induces intracellular imbalance of biothiols levels in NCM460 colonic cells to trigger intestinal inflammation in mice. Toxicol. Lett..

[10] Vally H. (2001). Role of sulfite additives in wine induced asthma: Single dose and cumulative dose studies. Thorax.

[11] Maiti B.K. (2022). Cross-talk Between (Hydrogen)Sulfite and Metalloproteins: Impact on Human Health. Chem. Eur. J..

[12] Kalimuthu P., Tkac J., Kappler U., Davis J.J., Bernhardt P.V. (2010). Highly Sensitive and Stable Electrochemical Sulfite Biosensor Incorporating a Bacterial Sulfite Dehydrogenase. Anal. Chem..

[13] Zeng R.-F., Lan J.-S., Wu T., Liu L., Liu Y., Ho R.J.Y., Ding Y., Zhang T. (2020). A novel mitochondria-targetted near-infrared fluorescent probe for selective and colorimetric detection of sulfite and its application in vitro and vivo. Food Chem..

[14] Devadas B., Sivakumar M., Chen S.M., Cheemalapati S. (2015). An electrochemical approach: Switching Structures of rare earth metal Praseodymium hexacyanoferrate and its application to sulfite sensor in Red Wine. Electrochim. Acta.

[15] Amatatongchai M., Sroysee W., Chairam S., Nacapricha D. (2015). Simple flow injection for determination of sulfite by amperometric detection using glassy carbon electrode modified with carbon nanotubes–PDDA–gold nanoparticles. Talanta.

[16] Ashmore P.L., Valdez F., Harbertson J.F., Boulton R.B., Collins T.S. (2023). Rapid determination of free sulfur dioxide in wine and cider by capillary electrophoresis. J. Chromatogr. A.

[17] Zhao L., Zhou J., Zhou J., Lin X., Huang K., Jiang X., Yu H., Xiong X. (2022). A microplasma converter-based spectrophotometry and visual colorimetry for nonchromatographic speciation analysis of H_2_S/SO_2_ or S^2−^/SO_3_^2−^ in environmental water samples. Microchem. J..

[18] McFeeters R.F., Barish A.O. (2003). Sulfite analysis of fruits and vegetables by high-performance liquid chromatography (HPLC) with ultraviolet spectrophotometric detection. J. Agric. Food. Chem..

[19] Long L., Han Y., Yuan X., Cao S., Liu W., Chen Q., Wang K., Han Z. (2020). A novel ratiometric near-infrared fluorescent probe for monitoring cyanide in food samples. Food Chem..

[20] Gan Z., Hu X., Xu X., Zhang W., Zou X., Shi J., Zheng K., Arslan M. (2021). A portable test strip based on fluorescent europium-based metal–organic framework for rapid and visual detection of tetracycline in food samples. Food Chem..

[21] Long L., Yuan F., Yang X., Ruan P., Chen X., Li L., He D., Yang S., Yang Y., Wang K. (2022). On-site discrimination of biothiols in biological fluids by a novel fluorescent probe and a portable fluorescence detection device. Sensor. Actuat. B-Chem..

[22] He D., Chen X., Yang S., Yang Y., Wang Y., Zhang R., Wang K., Qian J., Long L. (2023). A sensitive colorimetric and near-infrared fluorescent probe for tracing slight pH variation in food samples. ACS Food Sci. Technol..

[23] Chen G., Zhou W., Zhao C., Liu Y., Chen T., Li Y., Tang B. (2018). Rationally optimized fluorescent probe for imaging mitochondrial SO_2_ in HeLa cells and zebrafish. Anal. Chem..

[24] Yin C., Li X., Yue Y., Chao J., Zhang Y., Huo F. (2017). A new fluorescent material and its application in sulfite and bisulfite bioimaging. Sensor. Actuat. B-Chem..

[25] Sun Y.-Q., Wang P., Liu J., Zhang J., Guo W. (2012). A fluorescent turn-on probe for bisulfite based on hydrogen bond-inhibited C=N isomerization mechanism. Analyst.

[26] Wang C., Feng S., Wu L., Yan S., Zhong C., Guo P., Huang R., Weng X., Zhou X. (2014). A new fluorescent turn-on probe for highly sensitive and selective detection of sulfite and bisulfite. Sensor. Actuat. B-Chem..

[27] Yue L., Huang H., Song W., Lin W. (2022). A near-infrared endoplasmic reticulum-targeted fluorescent probe to visualize the fluctuation of SO_2_ during endoplasmic reticulum stress. Chem. Eng. J..

[28] Zhang H., Xue S., Feng G. (2016). A colorimetric and near-infrared fluorescent turn-on probe for rapid detection of sulfite. Sensor. Actuat. B-Chem..

[29] Li G., Chen Y., Wang J., Lin Q., Zhao J., Ji L., Chao H. (2013). A dinuclear iridium(iii) complex as a visual specific phosphorescent probe for endogenous sulphite and bisulphite in living cells. Chem. Sci..

[30] Tavallali H., Deilamy-Rad G., Parhami A., Lohrasbi S. (2016). A novel and simple fluorescent and colorimetric primary chemosensor based on Congo-Red for sulfite and resultant complex as secondary fluorescent chemosensor towards carbonate ions: Fluorescent probe mimicking INHIBIT logic gate. Talanta.

[31] Chen X., Chen Q., He D., Yang S., Yang Y., Qian J., Long L., Wang K. (2022). Mitochondria targeted and immobilized ratiometric NIR fluorescent probe for investigating SO_2_ phytotoxicity in plant mitochondria. Sensor. Actuat. B-Chem..

[32] Zhang W., Liu T., Huo F., Ning P., Meng X., Yin C. (2017). Reversible ratiometric fluorescent probe for sensing bisulfate/H_2_O_2_ and its application in zebrafish. Anal. Chem..

[33] Chen X., He D., Shentu J., Yang S., Yang Y., Wang Y., Zhang R., Wang K., Qian J., Long L. (2023). Smartphone-assisted colorimetric and near-infrared ratiometric fluorescent sensor for on-spot detection of H_2_O_2_ in food samples. Chem. Eng. J..

[34] Chen Q., Liu W., Han Y., Li L., Yuan F., Long L., Wang K. (2020). Accurately monitoring of sulfur dioxide derivatives in agricultural crop leaf tissues by a novel sensing system. Sensor. Actuat. B-Chem..

[35] Long L., Liu W., Ruan P., Yang X., Chen X., Li L., Yuan F., He D., Huang P., Gong A. (2022). Visualizing the Interplay of Lipid Droplets and Protein Aggregates During Aging via a Dual-Functional Fluorescent Probe. Anal. Chem..

[36] Li L., He D., Chen X., Yang S., Yang Y., Gong A., Wang K., Qian J., Long L. (2023). pKa-Tunable fluorescent probes for visualizing minor pH fluctuations in different subcellular organelles and in vivo. Dye. Pigm..

[37] Bi X., Li L., Luo L., Liu X., Li J., You T. (2022). A ratiometric fluorescence aptasensor based on photoinduced electron transfer from CdTe QDs to WS_2_ NTs for the sensitive detection of zearalenone in cereal crops. Food Chem..

[38] Long L., Huang M., Wang N., Wu Y., Wang K., Gong A., Zhang Z., Sessler J.L. (2018). A mitochondria-specific fluorescent probe for visualizing endogenous hydrogen cyanide fluctuations in neurons. J. Am. Chem. Soc..

[39] Peng X., Yang Z., Wang J., Fan J., He Y., Song F., Wang B., Sun S., Qu J., Qi J. (2011). Fluorescence ratiometry and fluorescence lifetime imaging: Using a single molecular sensor for dual mode imaging of cellular viscosity. J. Am. Chem. Soc..

[40] Hu X., Shi J., Shi Y., Zou X., Tahir H.E., Holmes M., Zhang W., Huang X., Li Z., Xu Y. (2019). A dual-mode sensor for colorimetric and fluorescent detection of nitrite in hams based on carbon dots-neutral red system. Meat Sci..

[41] Liang N., Hu X., Li W., Mwakosya A.W., Guo Z., Xu Y., Huang X., Li Z., Zhang X., Zou X. (2021). Fluorescence and colorimetric dual-mode sensor for visual detection of malathion in cabbage based on carbon quantum dots and gold nanoparticles. Food Chem..

[42] Zhu A., Ali S., Jiao T., Wang Z., Xu Y., Ouyang Q., Chen Q. (2023). Facile synthesis of fluorescence-SERS dual-probe nanocomposites for ultrasensitive detection of sulfur-containing gases in water and beer samples. Food Chem..

