# Supplementary material for: Construction of a Colorimetric and Near-Infrared Ratiometric Fluorescent Sensor and Portable Sensing System for On-Site Quantitative Measurement of Sulfite in Food
Source: Foods. 2024 Jun 4;13(11):1758. doi: 10.3390/foods13111758 (PMC11171829; doi:10.3390/foods13111758)
Supplement: Supplementary file 1 [file foods-13-01758-s001.zip › foods-3026984-supplementary.pdf]

Supporting information for

Construction of a Colorimetric and Near-infrared Ratiometric Fluorescent Sensor and Portable Sensing System for On-site Quantitatively Measuring Sulfite in Food

Xiaodong Chen,[1] Chenglu Zhao,[2] Qiwei Zhao,[2] Yunfei Yang,[2] Sanxiu Yang,[2] Rumeng Zhang,[2] Yuqing Wang,[2] Kun Wang,[1] Jing Qian,[2] Lingliang Long[2]*

[1] Key Laboratory of Modern Agricultural Equipment and Technology (Ministry of Education), Jiangsu University, Zhenjiang, Jiangsu 212013 (P. R. China).

[2] School of Chemistry and Chemical Engineering, Jiangsu University, Zhenjiang, Jiangsu 212013 (P. R. China).

Email: longlingliang@ujs.edu.cn.

Table of contents

Apparatus	S3
Scheme S1	S3
Synthesis of compound 1	S3
Synthesis of compound 2	S4
Synthesis of compound 3	S4
Preparation of the testing solution	S4
Determination of the detection limit	S4
Computational details	S5
Cell Culture	S5
Monitoring of sulfite in living cells by fluorescence imaging	S5
Monitoring of sulfite in zebrafish by fluorescence imaging	S5
Cultivation of lettuce	S5
Monitoring of sulfite in lettuce leaf tissues by fluorescence imaging	S5
Monitoring of sulfite in lettuce stem tissues by fluorescence imaging	S6
Preparation of portable sensing system	S6
On-site quantitative measurement of sulfite in water by sensor CY in combination with self-made portable sensing system	S6
Cultivation of tomato plants	S7
Figure S1	S7
Figure S2	S8
Figure S3	S8
Figure S4	S9
Table S1	S9
References	S10

Apparatus

^{1}H and ^{13}C nuclear magnetic resonance (NMR) spectra were measured on a Bruker Avance 400 spectrometer operating at 400 MHz and 100 MHz respectively. ESI mass spectrometry were measured on a Thermo Scientific LXQ Spectrometer. Absorption spectra were conducted on a SHIMADZU UV-2450 spectrometer. Fluorescence emission spectra were conducted on a Photon Technology International (PTI) Quantamaster fluorometer with 10 nm excitation and emission slit widths. The pH measurements were made on a pH-3c digital pH-meter (Shanghai ShengCi Device Works, Shanghai, China) with a combined glass-calomel electrode. Fluorescence imaging were conducted on a Leica TCS SP5 II laser confocal scanning microscope. Melting points were tested on a microscopy melting point apparatus (Beijing Taike XT-4).

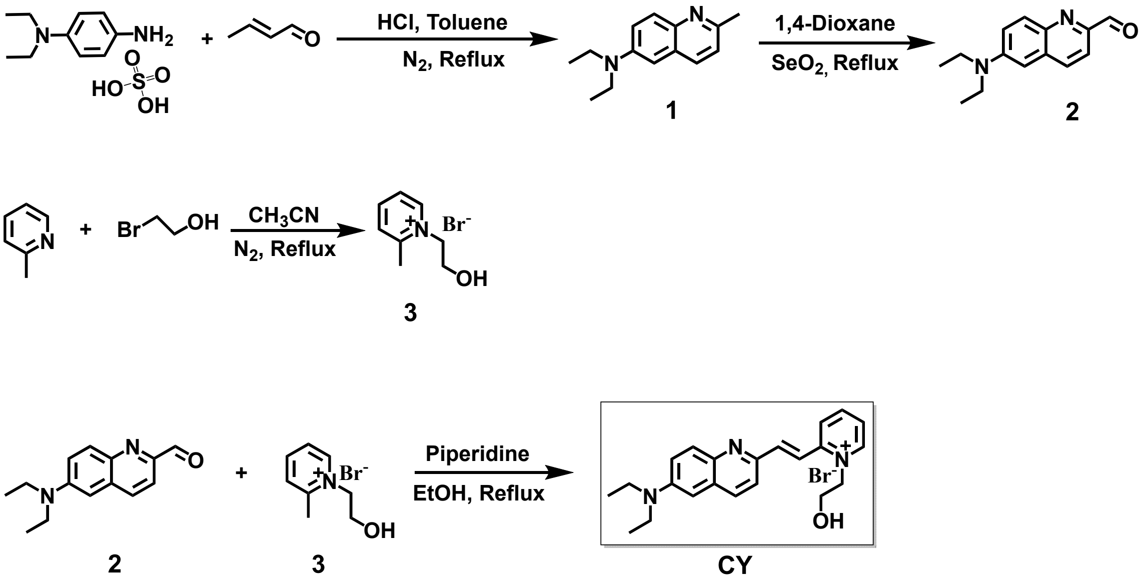

Scheme S1. The synthetical procedures for compound **CY**.

Synthesis of compound 1

N,N-Diethyl-p-phenylenediamine sulfate (1.5 g, 5.72 mmol) was dissolved in concentrated hydrochloric acid (4 mL) and water (1 mL). And then, crotonaldehyde (940 μL, 11.44 mmol) and toluene (1.5 mL) were added. Under N_2 atmosphere, the resulting mixture was heated to reflux for three hours. After cooling to room temperature, the organic phase was extracted three times with dichloromethane. The resulting organic phase was further washed with sodium hydroxide solution. The organic phase was evaporated under reduced pressure, and the residue was purified by column chromatography (dichloromethane: methanol = 100:1, v/v) to obtain bright yellow solid as compound **2** (723 mg, yield: 58.8 %). ^{1}H NMR ($CDCl_3$, 400 MHz), δ (ppm): 7.83 (m, 2H), 7.26 (m, 1H), 7.12 (d, J = 8.40 Hz, 1H), 6.73 (d, J = 2.80 Hz, 1H), 3.44 (q, J = 7.20 Hz, 4H), 2.65 (s, 3H), 1.12 (t, J = 7.20 Hz, 6H); MS (m/z): 215.49 $[M+H]^{+}$.

Synthesis of compound 2

SeO_2 (155 mg, 1.40 mmol) was dispersed in 15 mL of 1,4-dioxane. The resulting mixture was heated to reflux for 30 min. And then, compound **1** (150 mg, 0.70 mmol) was added to the mixture. The resulting solution was continued to be stirred and refluxed for 4 h. After the mixture cooling to room temperature, the organic phase was obtained by filtration. The organic phase was removed under reduced pressure, and the residue was purified by column chromatography (dichloromethane: methanol = 80:1, v/v) to obtain compound **3** as orange solid (76.80 mg, yield: 48%). ^1H NMR ($CDCl_3$, 400 MHz), δ (ppm): 10.11 (s, 1H), 8.04 (d, J = 9.6 Hz, 1H), 7.96 (d, J =8.8 Hz, 1H), 7.87 (d, J = 8.8 Hz, 1H), 7.38 (m, 1H), 6.74 (d, J =3.2 Hz, 1H), 3.55 (q, J = 7.2 Hz, 4H), 1.27 (t, J = 7.2 Hz, 6H); MS (m/z): 229.46 $[M+H]^+$.

Synthesis of compound 3

Under N_2 atmosphere, 4-methylpyridine (500 mg, 5.37mmol) and 2-Bromoethanol (2.01 g, 16.11 mmol) were dissolved in CH_3CN (3 mL). And then, the mixture was heated to reflux for 18 h. After cooling to room temperature, the yellow precipitate was filtered, washed with toluene, and dried in vacuo to afford compound **3** (259.6 mg, 22.1%), which was used directly for the next step without further purification.

Preparation of the testing solution

An appropriate amount of sensor **CY** was dissolved in DMSO to afford sensor **CY** (5×10^{-4} M) stock solution. Analyte stock solution (1×10^{-3} M) was obtained by dissolving a certain amount of analyte in water. The assay solution of the sensor **CY** (5 μM) was obtained by transferring 0.05 mL **CY** stock solution, 1.95 mL DMSO and a certain amount of each analyte stock into a 5.0 mL volumetric flask. Subsequently, the mixed solution was diluted to the mark with 10 mM potassium phosphate buffer (pH = 7.4). The obtained solution was sufficiently shaken and kept at room temperature for 5 min before measuring the fluorescence spectra.

Determination of the detection limit

The detection limit was calculated according to a method used in the literature. The fluorescence emission spectrum of sensor **CY** was measured five times and the standard deviation of a blank measurement was achieved. The ratio of fluorescence emission intensities (I_{447} / I_{692}) were plotted as a concentration of sulfite. The detection limit was calculated using the following equation:[1]

$$\text{Detection limit} = 3\,\sigma / k$$

Where σ is the standard deviation of blank measurement, k is the slope between the ratio of fluorescence emission intensities (I_{447} / I_{692}) versus sulfite concentration.

Computational details

The calculations were performed with Gaussian 09 program [2]. Becke's three-parameter and Lee-Yang-Parr hybrid functional (B3LYP) [3-5] and a 6-31+G(d,p) basis set [6] were used. The geometry of sensor **CY** and compound **CY-Sulfite** was optimized by density functional theory (DFT). The frontier molecular orbitals of **CY** and **CY-Sulfite** and the energy profiles were shown in Figure 3c.

Cell Culture

Caki-1 cells were seeded in 35-mm glass-bottomed culture dishes in Dulbecco's modified Eagle's medium (DMEM) supplemented with 10% fetal bovine serum and incubated in a humidified incubator containing 5% CO_2 in air at 37 °C.

Monitoring of sulfite in living cells by fluorescence imaging

The Caki-1 cells were stained with sensor **CY** (10 μM) for 30 min. After washing with PBS buffer three times, the cells were further treated with different concentration of sulfite (0, 100, 200, 300 μM) for 30 min. After that, fluorescence imaging was conducted on a Leica TCS SP5 II laser confocal scanning microscope with an objective lens (×20). The bule fluorescence channel was recorded at 410-450 nm with excitation at 405 nm. The NIR fluorescence channel was recorded at 680-700 nm with excitation at 543 nm.

Monitoring of sulfite in zebrafish by fluorescence imaging

Zebrafish were obtained as a gift from School of the Environment and Safety Engineering, Jiangsu University. The 5-day-old zebrafish were cultured at 28 ºC under alternating conditions of 12 h of daylight and 12 h of darkness in E3 embryo culture medium in 6-well plates.

Then, the zebrafish was stained with sensor **CY** (10 μM) in E3 embryo culture for 30 min. After washing with PBS buffer for three times, the zebrafish were further incubated with 0 μM or 200 μM sulfite for 30 min. Subsequently, fluorescence imaging was conducted on a Leica TCS SP5 II laser confocal scanning microscope with an objective lens (×20). The bule fluorescence channel was recorded at 410-450 nm with excitation at 405 nm. The NIR fluorescence channel was recorded at 680-700 nm with excitation at 543 nm.

Cultivation of lettuce

The seeds of lettuce were germinated on a wet gauze and kept in a wet state. After 2-3 days, the sprouted seeds were transferred to perlite for hydroponics. During the period of hydroponics, 25% Hoagland nutrient solution was supplied. One week later, the seedlings were transplanted into nutrient soil, and set in a greenhouse under an aspersion irrigation.

Monitoring of sulfite in lettuce leaf tissues by fluorescence imaging

The lettuce leaves were sprayed with different concentration of sulfite (0, 50, 100 and 300 μM) every day. After 5 days, the lettuce leaves were cut into thin slices. And then, the lettuce leaf slices were stained with sensor **CY** (10 μM) solution for 30 min.

Fluorescence imaging was conducted on a Leica TCS SP5 II laser confocal scanning microscope with an objective lens (×20). The bule fluorescence channel was recorded at 410-450 nm with excitation at 405 nm. The NIR fluorescence channel was recorded at 680-700 nm with excitation at 543 nm.

Monitoring of sulfite in lettuce stem tissues by fluorescence imaging

The lettuces were cultivated in nutrient soil with incremental amounts of sulfate (0, 50, 100 and 150 mM) for 7 days. After that, the lettuce stems were cut into thin slices. The lettuce stem slices were stained with sensor **CY** (10 μM) solution for 30 min. Fluorescence imaging was conducted on a Leica TCS SP5 II laser confocal scanning microscope with an objective lens (×20). The bule fluorescence channel was recorded at 410-450 nm with excitation at 405 nm. The NIR fluorescence channel was recorded at 680-700 nm with excitation at 543 nm.

Preparation of portable sensing system

In order to accomplish on-site quantitative measurement of sulfite content in food samples, a simple and portable sensing system was prepared. As illustrated in Figure 7A and Figure S3, the portable sensing system includes a dark box, petri dish, a white LED light as excitation source for colorimetric detection, a 480-485 nm LED light as excitation source for red fluorescence detection, a 380-385 nm LED light as excitation source for blue fluorescence detection, 420-480 nm and 680-700 nm optical filter, and a smartphone camera.

On-site quantitative measurement of sulfite in water by sensor CY in combination with self-made portable sensing system

Sulfite (Na_2SO_3) aqueous solutions at the concentration of 0, 30, 60, 90, 120, 150, 180, 210 and 240 μM were prepared. And then, 3 mL sulfite aqueous solution was added to a 5 mL volumetric flask containing 0.2 mL CY stock solution (5×10^{-4} M). Subsequently, the mixed solution was diluted to the mark with DMSO. Sensor **CY** (20 μM) were treated with these sulfite aqueous solutions for 30 min. And then, the solution (2 mL) was transferred to the petri dish (35 mm). For the colorimetric detection, the white LED light was used as excitation source. And the colorimetric images were directly captured by smartphone camera. After analyzed by Photoshop software, the R/(G+B) ratios (red intensity / (green intensity + blue intensity)) were obtained.

For fluorescence detection, the 380-385 nm LED light was used as excitation source for blue fluorescence detection. The blue fluorescence images were captured by the smartphone camera through a 420-480 nm optical filter. The 480-485 nm LED light was used as excitation source for red fluorescence detection. The red fluorescence images were captured by the smartphone camera through a 680-700 nm optical filter. After analyzed by Photoshop software, the blue and red fluorescence intensity ratios (B/R) were obtained.

Cultivation of tomato plants

The seeds of tomato were germinated on a wet gauze and kept in a wet state. After 2-3 days, the sprouted seeds were transferred to perlite for hydroponics. During the period of hydroponics, 25% Hoagland nutrient solution was supplied. One week later, the seedlings were transplanted into nutrient soil, and set in a greenhouse under an aspersion irrigation.

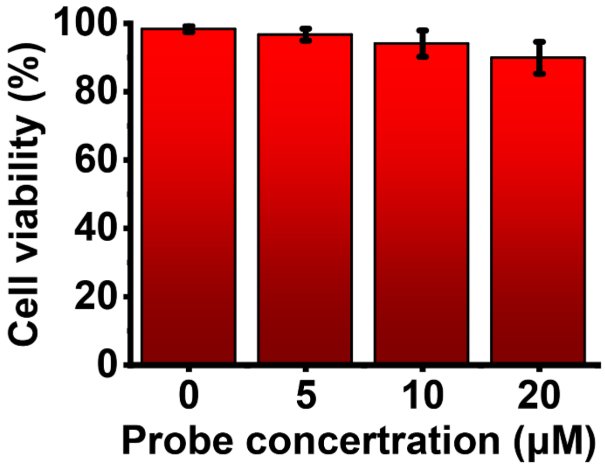

Figure S1. MTT results of Caki-1 cells viabilities after incubation with different concentration of sensor **CY** for 24 h. Data are expressed as mean ± SD (experiment times n = 3).

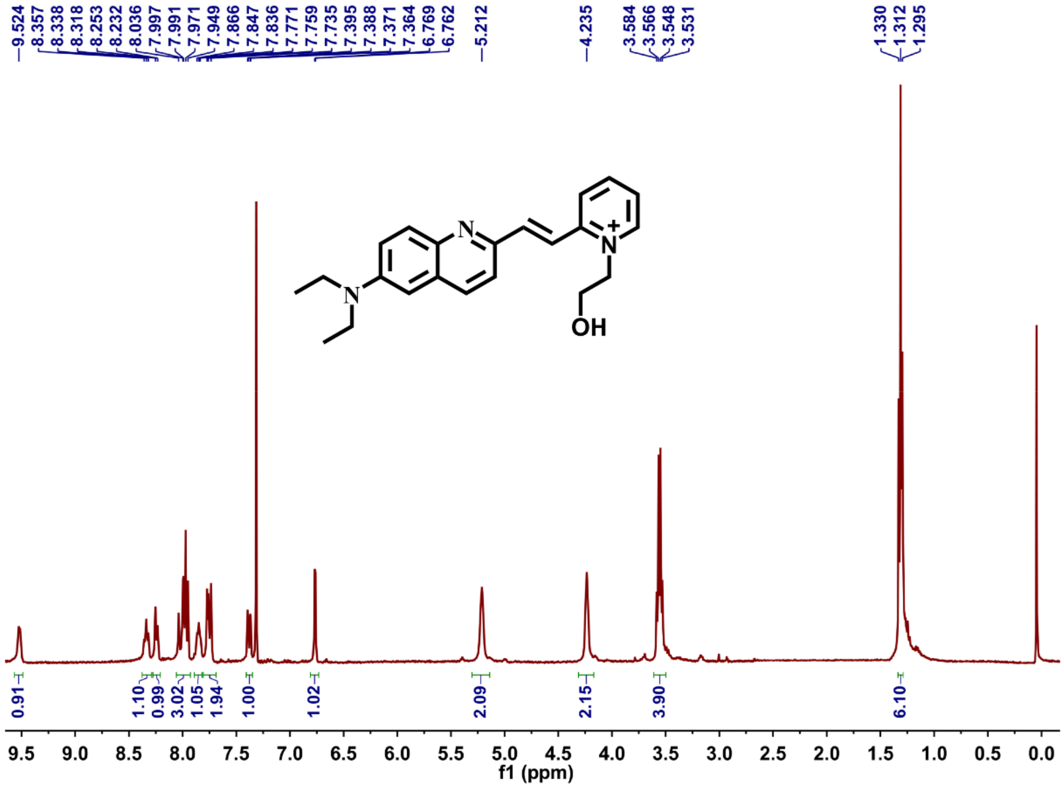

Figure S2. The ^{1}H NMR spectra of **CY** recorded in $CDCl_3$.

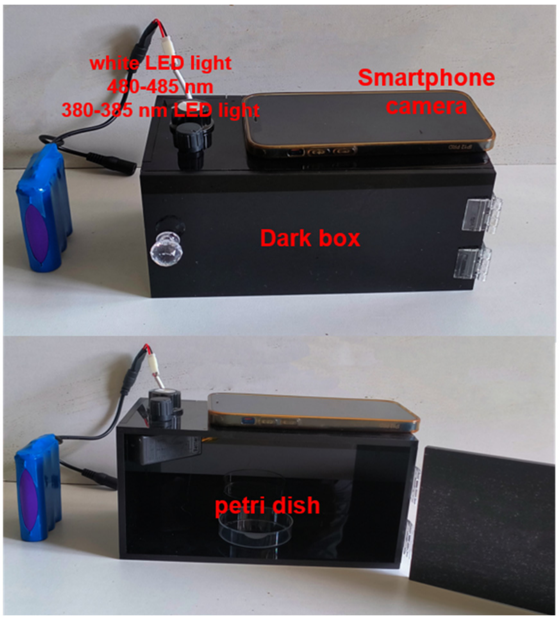

Figure S3. The photo of self-made portable sensing system.

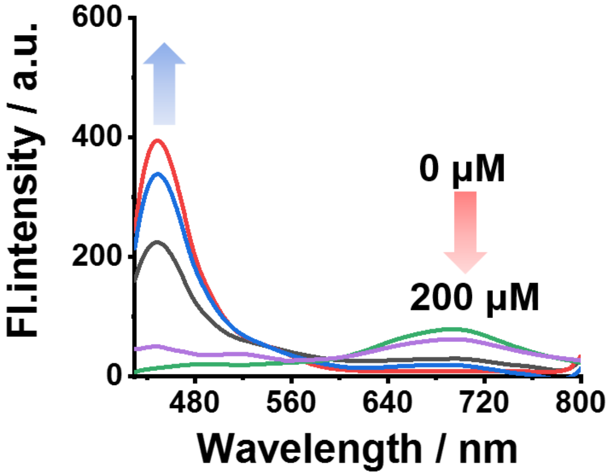

Figure S4 The fluorescence spectra (λ_{ex} = 410 nm) of sensor **CY** (5 μM) treated with increasing amounts of sulfite (0-200 μM).

Table S1 Comparison of the fluorescent sensors for sulfite detection in food analysis

Reference	Selectivity	Linear range	Response time	Sensitivity (LOD)	Portability
[7]	HSO_3^- / H_2O_2	0-150 μM	1 min	0.187 μM	NRD
[8]	Sulfite	0-9.5 μM	4.5 s	24 nM	NRD
[9]	HSO_3^- / SO_3^{2-}	5-30 μM	1 min	9.9 nM	NRD
[10]	HSO_3^- / SO_3^{2-}	2-16 μM	1 min	68 nM	NRD
[11]	H_2S / SO_2	200-800 μM	30 min	77.08 μM	NRD
[12]	HSO_3^- / SO_3^{2-} and viscosity	0.09-50 μM	30 min	0.09 μM	NRD
[13]	HSO_3^-	0-200 μM	N.P.	0.42 μM	Yes
[14]	HSO_3^-	0-24 μM	50 s	1.2 μM	NRD
[15]	Sulfite	0-20 μM	6 s	31.9 nM	Yes
[16]	SO_3^- / HSO_3^-	3.13-200 μM	30 min.	0.46 μM	NRD
This wok	Sulfite	0.203-90 μM	90 s	0.061 μM	Yes

N.P.: Not Provided, NRD: No related data presented.

References

1. B. Zhu, C. Gao, Y. Zhao, C. Liu, Y. Li, Q. Wei, Z. Ma, B. Du, X. Zhang, A 4-hydroxynaphthalimide-derived ratiometric fluorescent chemodosimeter for imaging palladium in living cells. *Chem Commun (Camb)* **2011**, 47,8656-8658.

2. Gaussian 09, Revision D.01, M.J. Frisch, G.W. Trucks, H.B. Schlegel, G.E. Scuseria, M.A. Robb, J.R. Cheeseman, G. Scalmani, V. Barone, B. Mennucci, G.A. Petersson, H. Nakatsuji, M. Caricato, X. Li, H.P. Hratchian, A.F. Izmaylov, J. Bloino, G. Zheng, J.L. Sonnenberg, M. Hada, M. Ehara, K. Toyota, R. Fukuda, J. Hasegawa, M. Ishida, T. Nakajima, Y. Honda, O. Kitao, H. Nakai, T. Vreven, J.A. Montgomery, Jr., J.E. Peralta, F. Ogliaro, M. Bearpark, J.J. Heyd, E. Brothers, K.N. Kudin, V.N. Staroverov, T. Keith, R. Kobayashi, J. Normand, K. Raghavachari, A. Rendell, J.C. Burant, S.S. Iyengar, J. Tomasi, M. Cossi, N. Rega, J.M. Millam, M. Klene, J.E. Knox, J.B. Cross, V. Bakken, C. Adamo, J. Jaramillo, R. Gomperts, R.E. Stratmann, O. Yazyev, A.J. Austin, R. Cammi, C. Pomelli, J.W. Ochterski, R.L. Martin, K. Morokuma, V.G. Zakrzewski, G.A. Voth, P. Salvador, J.J. Dannenberg, S. Dapprich, A.D. Daniels, O. Farkas, J.B. Foresman, J.V. Ortiz, J. Cioslowski, D.J. Fox, Gaussian, Inc., Wallingford CT, **2013**.

3. A.D. Becke, Density-functional thermochemistry. III. The role of exact exchange. *J. Chem. Phys.* **1993**,98,5648-5652.

4. C. Lee, W. Yang, R.G. Parr, Development of the Colle-Salvetti correlation-energy formula into a functional of the electron density. *Phys. Rev.* B **1988**,37,785.

5. P. Stephens, F. Devlin, C. Chabalowski, M.J. Frisch, Ab initio calculation of vibrational absorption and circular dichroism spectra using density functional force fields. *J. Chem. Phys.* **1994**,98,11623-11627.

6. V.A. Rassolov, M.A. Ratner, J.A. Pople, P.C. Redfern, L.A. Curtiss, 6-31G* basis set for third-row atoms. *J. Comput. Chem.* **2001**,22,976-984.

7. Huang Y., Li Y., Huang X., Tang L., Yan X., A novel "AIE+ESIPT" mechanism-based fluorescent probe for visual alternating recognition of HSO_3^-/H_2O_2 and its HSO_3^- detection in food samples, *Dyes Pigm.* **2024**,*222*,111901.

8. Jiang L., Chen T., Song E., Fan Y., Min D., Zeng L., Bao G.-M., High-performance near-infrared fluorescence probe for fast and specific visualization of harmful sulfite in food, living cells, and zebrafish, *Chem. Eng. J.* **2022**,*427*,131563.

9. Li F., Zhai S.-M., Xian-Yu J.-J., Zhao B.-X., Lin Z.-M., NBD-based colorimetric and ratiometric fluorescent probe in NIR for bisulfite, *Talanta* **2024**,*271*,125684.

10. Li F., Zhang Y.-Y., Liu T.-Z., Wei B.-Y., Miao J.-Y., Zhao B.-X., Lin Z.-M., Water-soluble fluorescent probe for specific detection of SO_2 derivatives in food and cells, *Dyes Pigm.* **2023**,*219*,111655.

11. Li H., Liu Y., Wang Y., Li J., Li Y., Zhang G., Zhang C., Shuang S., Dong C., A near infrared fluorescence probe with dual-site for hydrogen sulfide and sulfur dioxide detection, *Spectrochim. Acta A Mol. Biomo.l Spectrosc.* **2024**,*305*,123523.

12. Liu F.-T., Jiang P.-F., Wang Y.-P., Zhao B.-X., Lin Z.-M., A ratiometric fluorescent probe based on the FRET platform for the detection of sulfur dioxide derivatives and viscosity, *Anal. Chim. Acta* **2024**,*1288*,342184.

13. Mi W., Shen T., Guo X., Liu X., Zhang M., Jia M., Ratiometric quantification and visual detection of sulfur dioxide residues using a coumarin-derived fluorescent probe, *Sensor. Actuat. B-chem.* **2023**,*395*,134459.

14. Shang Z., Liu J., Meng Q., Wang Y., Zhang C., Zhang Z., A near-infrared emitted fluorescence probe for the detection of biosulfite in live zebrafish, mouse and real food samples, *Methods* **2022**,*204*,47-54.

15. Yue C., Zeng L., Zhang D., Li K., Jiang L., Xie P., A practical chromogenic and fluorogenic dual-

mode sensing platform for rapid quantification of sulfite in food, *Food Chem.* **2024**,*440*,138183.
16. Zeng R.-F., Lan J.-S., Wu T., Liu L., Liu Y., Ho R.J.Y., Ding Y., Zhang T., A novel mitochondria-targetted near-infrared fluorescent probe for selective and colorimetric detection of sulfite and its application in vitro and vivo, *Food Chem.* **2020**,*318*,126358.
